# Deep learning methods allow fully automated segmentation of metacarpal bones to quantify volumetric bone mineral density

**DOI:** 10.1038/s41598-021-89111-9

**Published:** 2021-05-06

**Authors:** Lukas Folle, Timo Meinderink, David Simon, Anna-Maria Liphardt, Gerhard Krönke, Georg Schett, Arnd Kleyer, Andreas Maier

**Affiliations:** 1 Pattern Recognition Lab-Computer Science, Friedrich-Alexander Universität Erlangen-Nürnberg (FAU), Erlangen, Germany; 2 Department of Internal Medicine 3-Rheumatology and Immunology, FAU Erlangen-Nürnberg and Universitätsklinikum Erlangen, Erlangen, Germany; 3 Deutsches Zentrum für Immuntherapie, FAU Erlangen-Nürnberg and Universitätsklinikum Erlangen, Erlangen, Germany

**Keywords:** Rheumatic diseases, Computer science

## Abstract

Arthritis patients develop hand bone loss, which leads to destruction and functional impairment of the affected joints. High resolution peripheral quantitative computed tomography (HR-pQCT) allows the quantification of volumetric bone mineral density (vBMD) and bone microstructure in vivo with an isotropic voxel size of 82 micrometres. However, image-processing to obtain bone characteristics is a time-consuming process as it requires semi-automatic segmentation of the bone. In this work, a fully automatic vBMD measurement pipeline for the metacarpal (MC) bone using deep learning methods is introduced. Based on a dataset of HR-pQCT volumes with MC measurements for 541 patients with arthritis, a segmentation network is trained. The best network achieves an intersection over union as high as 0.94 and a Dice similarity coefficient of 0.97 while taking only 33 s to process a whole patient yielding a speedup between 2.5 and 4.0 for the whole workflow. Strong correlation between the vBMD measurements of the expert and of the automatic pipeline are achieved for the average bone density with 0.999 (Pearson) and 0.996 (Spearman’s rank) with $p < 0.001$ for all correlations. A qualitative assessment of the network predictions and the manual annotations yields a 65.9% probability that the expert favors the network predictions. Further, the steps to integrate the pipeline into the clinical workflow are shown. In order to make these workflow improvements available to others, we openly share the code of this work.

## Introduction

Hand bone loss is characteristic for chronic arthritis such as rheumatoid or psoriatic arthritis, which clinically manifests with pain, swelling and stiffness of the affected joints^1,2^. Hand joints are typically affected in arthritis^3^. Conventional radiographs (CR) are widely used to detect hand bone loss in arthritis^4^. However, an exact and early quantification of bone density and microstructure is not possible with conventional radiography due to the nature of plain images.

In contrast to conventional radiography, high resolution peripheral quantitative computed tomography (HR-pQCT) allows three-dimensional (3D) determination and quantification of the microarchitecture as well as volumetric bone mineral density (vBMD) of the hand joints in vivo with a resolution of 82 micrometer isotropic voxel size^5,6^. Subsequently, density parameters (mg hydroxylapatite/${\text {cm}}^3$) are available for the whole bone.

In the past years HR-pQCT has been used to determine and quantify the vBMD and microarchitecture in patients with arthritis, which has supported the early and differential diagnosis of the disease and evaluated the bone-protective effort of anti-rheumatic drugs^7,8^.

State-of-the-art (SOTA) assessment for vBMD determination of the hand using HR-pQCT technique is based on a semi-automatic segmentation of the metacarpal (MC) bone in the acquiried images^9^. Given the segmentation mask, the vBMD is calculated by the summation of corresponding bone voxels. However, the semi-automatic segmentation of up to 320 slices for each patient joint by an expert is a time-intensive process. Additionally, for patients with advanced stages of arthritis, the erosion of the cortical bone of the MCs poses challenges for the segmentation task as the differentiation of the bone from the surrounding tissue is difficult. For mild cases this leads to inter-operator precision errors of 3.7%^10^. However, for difficult cases, this situation can lead to inter-operator precision errors as high as 11.58% for the erosions^11^.

Advancements in the field of deep learning have enabled the application of neural networks to medical imaging tasks^12^. The automatic segmentation of bones in whole body computed tomography (CT) images demonstrates the ability of neural networks to generalize to new, unseen patient images for a compliated task^13^. Recent works transitioned from feasability studies to the examination of the benefits of using deep learning as an assisting tool in the daily routine of clinicians^14^.

The objectives of this work are to evaluate the potential of neural networks to replace the semi-automatic segmentation by an expert and to implement the automatic segmentation pipeline in the clinical workflow, both by comparing SOTA and the developed pipeline with respect to the accuracy of the segmentations, the correlation of vBMD results, and the time required to assess vBMD in arthritis patients.

## Results

**Figure 1:**
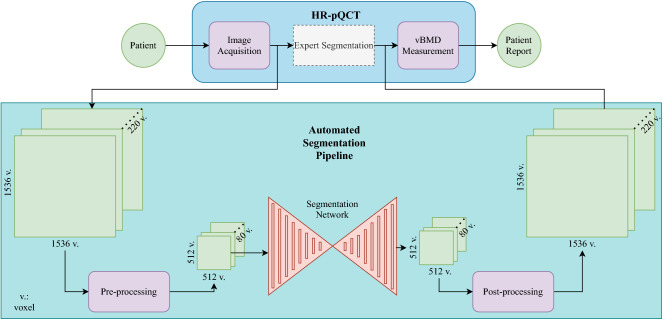
The pipeline to generate a patient report can be subdivided into the following steps: (1) image acquisition using the HR-pQCT scanner, (2) segmentation of the second MC in the volumes, (3) calculation of the vBMD using the segmentation masks. As the manual and the automatic pipeline only differ in the segmentation step, the automatic segmentation pipeline can be considered a drop-in replacement of the semi-automatic, time-intensive expert segmentation.

First, the performance of three neural network configurations is compared with the clinical expert’s manual segmentation using discriminative metrics. Subsequently, to evaluate the effect of the automatic pipeline in Fig. 1 using a neural network, the time requirements are compared to the manual workflow. Finally, the vBMD measurements obtained by the clinicians will be compared to the network-based automatic approach.

### Network configuration and performance

The three configurations compared in the following are

- 2D Segmentation network (2D U-Net) without pre-training,
- 2D Segmentation network (2D U-Net) with pre-training, and
- 3D Segmentation network (3D U-Net) without pre-training.

For the 3D U-Net, no compatible pre-trained weights were available due to the shallowness of the network. In order to avoid the random choice of good initialization parameters, for the networks without pre-training, each training is repeated three times. Each time the random seed is perturbed for all software frameworks used.

Apart from a selection of patient scans with few motion artifacts, the following criteria resulted in inclusion: patients provided consent to use the data and data was obtained during the clinical routine. Pregnant patients and patients under 18 were excluded. Note, that for pregnant patients no scans can be acquired. From the total number of 541 patient scans, 130 left hands and 411 right hands were assessed. The patient scans can be divided into 195 volumes with motion grade 1, 70 with motion grade 2, and 276 with motion grade 3. The age of the arthritis patients scanned, ranged from 18 to 85 years with a mean age of 53.8 and a standard deviation of 12.9. Slightly more female patients with 329 acquisitions than male patients with 212 acquisitions were acquired.

#### Segmentation performance assessment

The assessment of the quantitative performance is based on the comparison of the segmentation masks by the expert annotator and the predictions of the automatic segmentation pipeline. Based on these segmentation masks, a correlation analysis of the vBMD measurements was performed.

The accuracy reached by all configurations was as high as 0.999 making a detailed comparison of the configurations impossible. Therefore, more discriminative metrics such as the intersection over union (IOU), Dice coefficient, and area under the curve (AUC) were used. The 2D U-Net reached an IOU of 0.933. Switching to the 3D network, the value was improved by 0.004. The pre-trained U-Net reached an IOU of 0.943 (compare Table 1). As for the Dice coefficient, the 2D U-Net reached 0.965 followed by the 3D U-Net with 0.969, and the pre-trained 2D U-Net with 0.972. Finally, the 2D U-Net reached an AUC of 0.996, the 3D U-Net of 0.998, and the pre-trained 2D U-Net of 0.999.

**Table 1:** Comparison of the metrics on the test set consisting of 54 patients for the three network configurations.

Network	2D U-Net	3D U-Net	2D U-Net (pre-trained)
IOU	0.933 (0.009)	0.937 (0.003)	**0.943**
Dice	0.965 (0.006)	0.969 (0.002)	**0.972**
AUC	0.996 (0.005)	0.998 (0.001)	**0.999**

Mean values are followed by standard deviation in brackets. The 2D U-Net (pre-trained) is trained once, the two other configurations are trained three times with different random seeds. Best metrics are highlighted in bold font.

**Figure 2:**
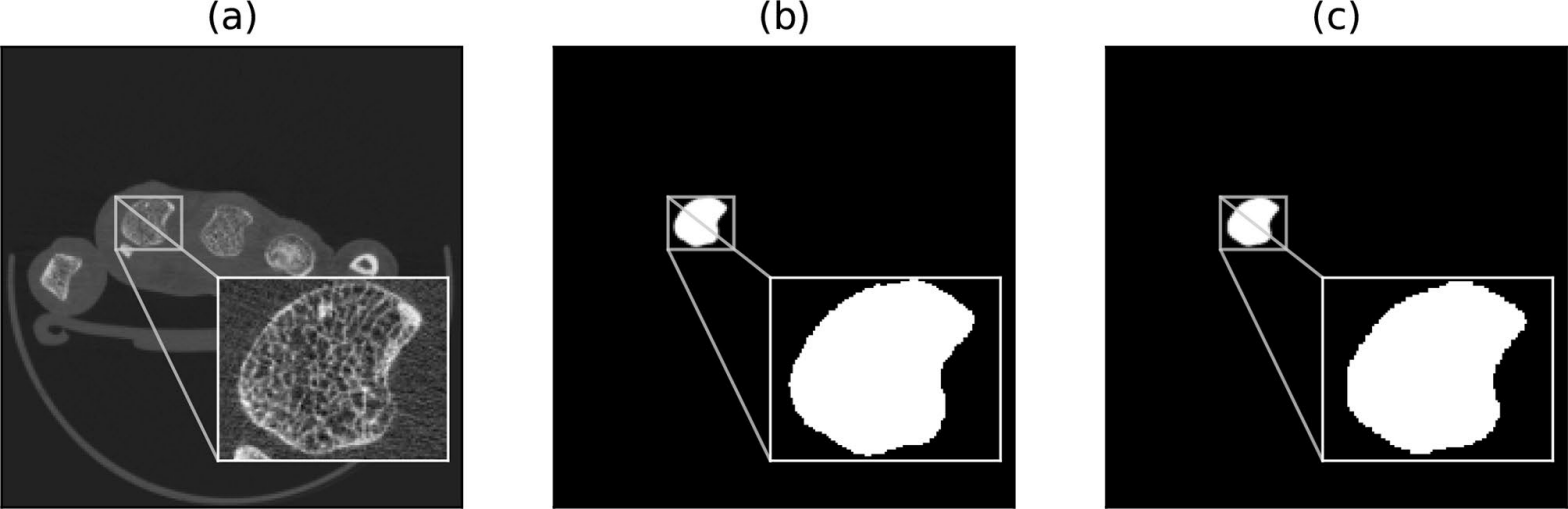
Overview of a slice of the HR-pQCT volume (**a**), the manual segmentation (**b**), and the network-based segmentation (**c**). Note, that the input to the network are the whole slices of the volume.

An exemplary input to the network and the corresponding manual segmentation is compared to the network-based segmentation in Fig. 2.

The segmentation of the second MC is an intermediate result of the whole pipeline and thus, to compare the final results of both pipelines, the vBMD measurements on a subset of the test-set (n = 30) are utilized.

The mean area of the slices for the manual pipeline was $122.22\,{\text {mm}}^2$ (SD 19.69) and $116.71\,{\text {mm}}^2$ (SD 19.03) for the automatic pipeline. The vBMD results of the manual pipeline for the average bone mineral density (D100) were in the range between 162.5 and 377.0 mg HA/${\text {cm}}^3$ with a mean of 279.32 mg HA/${\text{ cm }}^3$ (SD 42.80) and for the automatic pipeline between 147.6 and 363.9 mg HA/${\text{ cm }}^3$ with a mean of 265.29 mg HA/${\text{ cm }}^3$ (SD 43.18).

**Figure 3:**
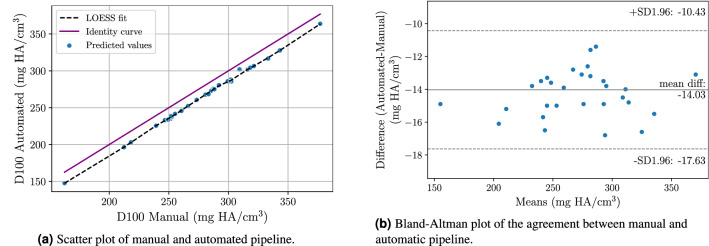
Correlation analysis of the average bone mineral density (D100) comparing the manual segmentations and the network-based segmentations.

Significant Pearson correlation for D100 with 0.999 ($p < 0.001$) was reached. Additionally, significant Spearman’s-rank for D100 with 0.996 ($p < 0.001$) was achieved. Additionally, an agreement of 0.947 for the intraclass correlation (ICC) could be achieved. The scatter plot in Fig. 3a demonstrates the correlation of the average bone mineral densities while the Bland-Altman^15^ plot in Fig. 3b allows for an analysis of the error between automated and manual pipeline.

#### Expert rating of network predictions

**Figure 4:**
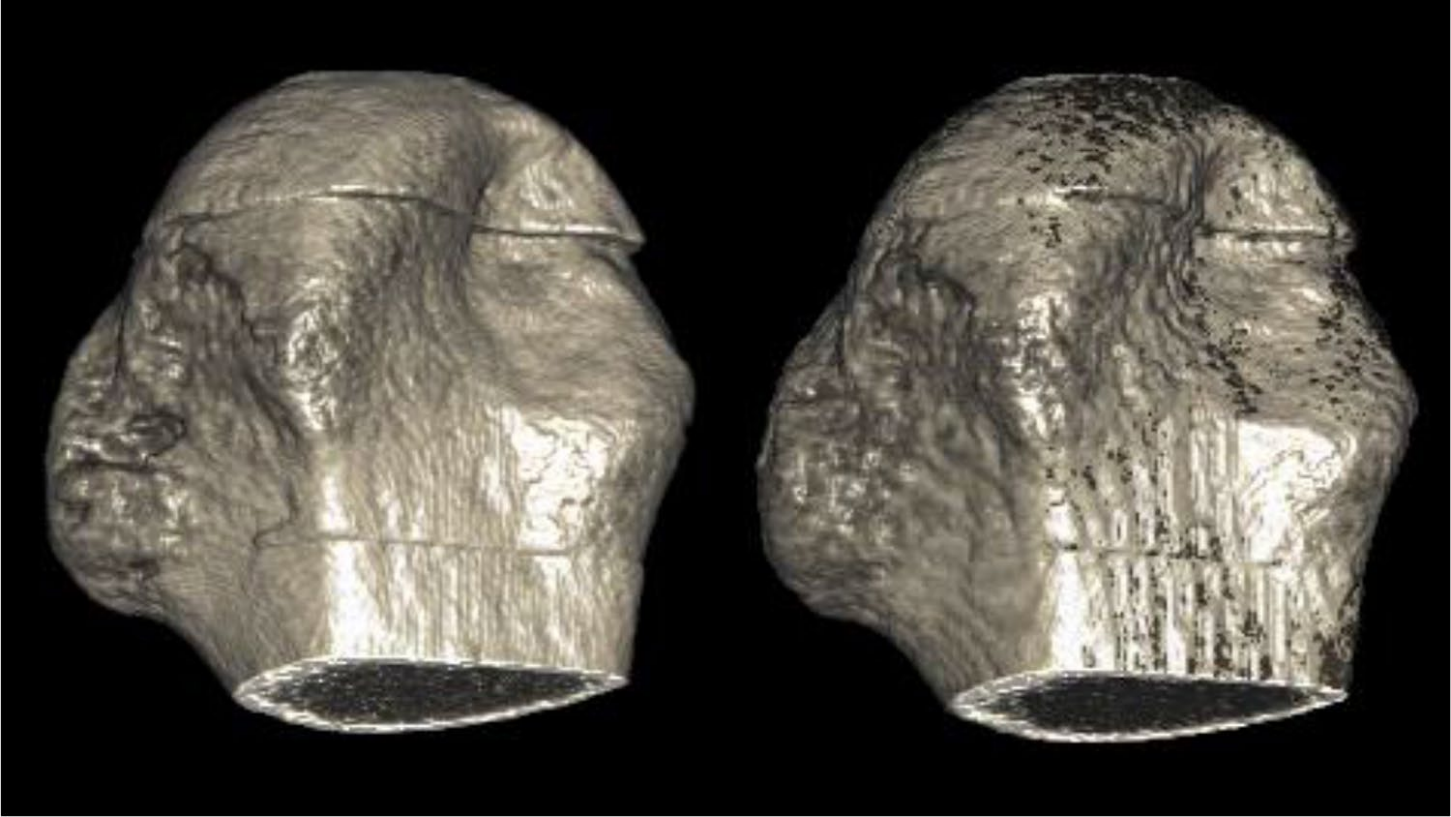
Volume rendering of the expert annotation (left) and the network prediction (right). The binary masks were multiplied with the HR-pQCT volume to yield the respective bone volumes. Note that the rendering of the prediction appears slightly degraded due to the up-sampling as part of the post-processing pipeline. The bones were rendered using MeVisLab version 3.1.0 (https://www.mevislab.de/).

In total, 49 of the 54 test cases were classified by an expert (technician with 3 years of experience) into three classes. In seven cases, none was chosen, as both contours were described as equally good. For the remaining 42 cases in 65.9% of the cases, the contour generated by the automatic segmentation pipeline was chosen as the preferred contour (Fig. 4).

### Time to measurement

In the following, all steps of the manual pipeline were compared with the fully automatic pipeline depicted in Fig. 1 with respect to the time requirements.

#### Image acquisition

The image acquisition was equal for the manual and the fully automatic pipeline. However, in order to compare the two pipelines, all the respective steps have to be considered for a complete comparison of the time requirements. The fixation and the scanning of the patient’s hand took 8.4 min^16^.

#### Segmentation

For the 2D U-Net, the network-based pipeline took on average 33.45 s (SD 0.08). The 3D U-Net slightly surpassed the 2D U-Net with an average of 32.98 s (SD 0.09). The comparison of the semi-automatic segmentation and the network-based segmentation is shown in Table 2 in the Segmentation row.

**Table 2:** Comparison of the gold standard manual pipeline and the automatic pipeline.

Pipeline type	Manual pipeline	Automated pipeline
Image acquisition (measurement time)	8.4 min	8.4 min
**Segmentation**	**15–30 min**^19^	**0.5 min**
vBMD measurement	1 min	1 min
Total time	24.4–39.4 min	9.9 min
**Speedup (with respect to manual pipeline)**	**1**	**2.5–4.0**

Manual and automatic pipeline only differ in the segmentation step. For the manual pipeline an expert has to annotate the patient’s slices semi-automatically while for the automatic pipeline, a neural network predicts the location of the second MC. Both network variants, the 2D U-Net and the 3D U-Net have similar run times for the segmentation task. Differences of time between manual pipeline and automated pipeline are highlighted in bold font.

#### vBMD calculation

The last step of the clinical workflow is the calculation of the volumetric bone mineral density. Similar to the image acquisition, this step did not differ for the automatic and the manual pipeline. Based on the segmentation from the previous pipeline step, the bone can be extracted by a multiplication of the acquired image and the segmentation mask^17^. Finally, the vBMD for the entire bone can be calculated using the intensity values of the calibrated images.

The calculation of the vBMD for the segmented bone consists of the summation of the corresponding voxels in the calibrated images. On average, this step takes 1 min^18^.

## Discussion

In this work, a fully automatic vBMD measurement pipeline was introduced. This pipeline extends previous work by the integration of the automatic segmentation pipeline into clinically used systems and the ability to yield the vBMD measurements for the joints of the patients^19–21^. The introduced pipeline succeeds the SOTA in terms of the time requirements and shows very high agreement with the expert-based measurements. Additionally, the dataset used to train and test the segmentation network is substantially larger than in previous works.

Prior research demonstrated the high interest in semi-automatic and fully automatic segmentation techniques of hand bones affected by arthritis leading to differential diagnostic and prognostic approaches^21,22^. Whittier et al. analyzed an automatic contouring algorithm for the radius and the tibia and compared the results with expert annotators^19^. They established that manual corrections by an expert are necessary for the algorithm used. Figueiredo et al. compared manual segmentations of erosions in the second and third MC with two semi-automatic approaches^21,22^. They proved the semi-automatic approaches to be very helpful and showed good agreement between the semi-automatic approaches. Valentinitsch et al. developed an automatic segmentation algorithm for the distal radius but not for the hand bones^20^. Based on a preselected region of interest, their algorithm outperformed the HR-pQCT manufacturer’s algorithms and achieved a Dice similarity coefficient of 0.90. Automated segmentation of wrist bones in magnetic resonance imaging (MRI) based on a dataset of 34 images was investigated by Wodarczyk et al.^23^. For the set of 34 images, they achieved very good performance and demonstrated the automatic detection of lesions for patients with RA. Finally, Lauze et al. showed that deep learning methods can be used to detect erosions in HR-pQCT images for a set of only three patients^21^.

The segmentation of the second MC of the acquired images is a crucial part in both pipelines. For the semi-automatic segmentation, this process requires the attention of an expert for a substantial period of time. Whittier et al. analyzed the contouring process of the tibia and the radius in HR-pQCT volumes with 168 slices^19^. This segmentation task is comparable to the segmentation of the second MC as the resolutions of the images are comparable and both tasks make use of a semi-automatic annotation tool. For difficult cases Whittier et al. reported an annotation time of 15–30 min for an expert, and 45 min for a beginner operator.

The 2.5–4 fold speedup of the introduced fully automatic segmentation pipeline compared to the SOTA approach yielded significant time savings, reduced work for the clinicians, and is observer-independent. As the introduced pipeline was not evaluated in a clinical trial, a control of the generated contour is necessary. This will reduce the speedup slightly. However, the most time demanding step of the automatic pipeline is the image acquisition. Thus, a review of the predicted contours will only have a minor impact upon the overall speedup. All technical obstacles that impose challenges to the introduction of an automatic vBMD measurement pipeline have been taken in this work. To further evaluate the approach, the pipeline will be assessed in longitudinal studies in the future. In order to achieve medical device approval and pass the regulatory affairs, a more detailed analysis of our method is required. First, the motion grading needs to be standardized across multiple clinical sites. Second, the area to be segmented has to be defined specifically to yield comparable results. Finally, a multi-center study has to be performed to assess the performance for the extended dataset.

The quantitative evaluation demonstrated the ability of the automatic segmentation pipeline to generalize well to new patients. Strong correlations between the manual pipeline and the automatic pipeline suggest a very good coherence between both pipelines. The Bland-Altman plot in Fig. 3b indicates a slight underestimation of the measurements for D100 by the network-based approach.

The qualitative evaluation yielded that the predictions of the network-based pipeline are favored over the expert annotations. This can be explained by the fact that the network produces very consistent results, while clinicians are typically subject to inter-, and intra-reader variability^24^. The extension of the automatic pipeline to other bones affected by arthritis might help to further improve the clinical value of the approach and has the potential to yield advanced robustness for the patients’ vBMD measurements.

Concerning the network’s input dimensionality, operating on single slices (2D) of the patient volume has the advantage that the memory consumption of the input data is drastically smaller. Subsequently, the depth of the network can be increased and thus, the network’s predictive capabilities increases given that enough training samples are available. Operating directly on the whole patient volume (3D) allows the network to make more consistent predictions along the slice direction as it can learn the variance of the data in this direction. However, the layers of the 3D networks have a higher memory consumption. Thus, the number of layers has to be greatly reduced when switching from 2D to 3D networks. As a side effect, this hinders the 3D network from remembering the training volumes (overfitting) and might as well lead to a better generalization to new patients.

Sampling the initial network parameters from a specific distribution with certain properties can lead to the discovery of new locally optimal network parameters using an optimization algorithm. However, low-level features such as edges and corners do not differ across different modalities extensively^25^. Thus, the parameters of a network trained on the task of classifying images to a set of classes such as cars, plants, and houses, can also be sensible for the segmentation of bones in HR-pQCT images^26^. These pre-trained networks typically have the advantage to converge faster to the optimum and thus, reduce the required time for training.

In conclusion, our work provides evidence that the neural-network-based segmentation approach can be fully integrated into the workflow of rheumatologists. While currently the introduced approach still requires human supervision and is not validated on a regulatory basis, the speedup compared to the expert-based approach and the very high agreement with the experts are promising results and motivate further steps towards the use in the clinical setting.

## Methods

### Dataset

Deep learning methods require a substantial amount of labeled data to achieve good performance and generalize well to new cases^27^. Due to privacy constraints and regulations in the medical context, this amount of data is typically hard to achieve. In the following, the image acquisition is described. Based on this, the dataset used to train the neural networks are analyzed in detail.

#### Image acquisition

HR-pQCT scans of joints from patients with different forms of arthritis (rheumatoid arthritis, psoriatic arthritis, osteoarthritis) that were acquired during a clinical routine visit between 2008 and 2018 were used for this analysis. These patients were seen at the Department of Internal Medicine 3 of the University Hospital Erlangen. Ethics approval to analyze the images was obtained from the ethics committee of Friedrich-Alexander-Universität (approval number 324_16 B) and patients gave informed consent. All experiments were performed in accordance with relevant guidelines and regulations. The image acquisition closely followed the procedure described in Simon et al^6^. Scans of the second MC joints of the dominant hand of patients were acquired with an isotropic voxel size of 82 $\upmu$m using an HR-pQCT scanner (XtremeCT 1, Scanco Medical, Brütisellen, Switzerland). To reduce patient motion, a custom hand holder was used during the acquisitions. As motion artifacts can greatly affect HR-pQCT outcome measures, all scans are routinely assessed for the motion grade (Score 1–5)^28^. Adapted from the clinical routine, acquisitions of motion grade greater than 3 were not included in the analysis. The X-ray tube was set to an effective energy of 60 kVp with a tube current of 900 $\upmu$A, and an integration time of 100 ms. This results in a patient dose of < 3 $\upmu$Sv for 111 slices. The scanning time for the second MC was 8.4 min and ranges between 200 and 320 slices, while the number of segmented images depends on the anatomy of the patient.

#### Statistics

In total, 541 patient volumes and the corresponding expert annotations (technician with 3 years of experience) were available. To evaluate the three network configurations, a test-set with 54 unseen patients was randomly picked from the total set of 541 patients. In order to test the performance of the whole automatic segmentation pipeline, the post-processing steps were also incorporated in the evaluation, leading to a comparison of automatic segmentation pipeline prediction and expert annotation at full resolution. During the qualitative performance assessment, 49 of the 54 patients from the test-set were picked randomly. For the comparison of the vBMD measurements, 30 patients were picked randomly from the test-set and evaluated using Pearson correlation, Spearman-rank, and Bland-Altman plots. P-values less than 0.05 were considered significant.

### Neural network

Based on the findings of the dataset, in this section, the parts related to the training of the neural networks are described. As depicted in Fig. 1, the automatic segmentation pipeline consists of the pre-processing of the HR-pQCT data, a forward pass through the network, and the post-processing to enable the subsequent vBMD measurement.

#### Pre-processing

As the training is based on the comparison of input volumes and expert annotations, the expert annotations were also pre-processed. The first step was the transformation of the expert annotator contour to a binary image (details and the commands used are described in the “Supplementary information”). Next, the expert annotation and the patient volume were aligned using the volume of interest information of the contour. The number of slices of the patient volume necessary for the later vBMD measurement was not constant for the second MC and changes with patient’ height. The expert annotator typically stoped annotation when the bone has a delta shape in the current slice. To enable a consistent training, all slices in the patient volume not annotated were dropped. Spatial dimensions of $1536 \times 1536$ voxels allow a very precise annotation. However, the internal representation of the volume or the individual slices take a considerable amount of video memory. Therefore, the volume was resized to $512 \times 512$ voxels and 80 slices for all patients. In order to reduce the variance of the data, left hands were flipped along the y-axis yielding a dataset of right hands. Finally, the intensity values of the patient volume were normalized using a zero mean, unit variance normalization based on the mean and variance of the training dataset.

#### Networks

The 2D segmentation model is based on Ronneberger et al.’s U-Net^29,30^. It features a ResNet34^31^ encoder with a depth of 5 and the following internal number of channels: 3, 64, 64, 128, 256, 512. The decoder is the standard U-Net decoder with 256, 128, 64, 32, and 16 channels. ReLU is used as the activation function. The pre-trained weights are based on the ImageNet dataset^32^.

The 3D segmentation model is an extension of the aforementioned 2D U-Net^33^. Just like the 2D U-Net the 3D U-Net has a depth of five. The number of channels starts with two in the first depth stage and is doubled for each subsequent stage. LeakyReLU^34^ is used as the activation function and in the first stage dropout with 60% probability is used.

The dataset was split into training set, validation set, and test set with a distribution of 70% (n = 378), 20% (n = 108), and 10% (n = 54), respectively. Patients with erosions were present in all three dataset splits. All networks were trained using the Adam optimizer with default values. The 2D segmentation models were trained with a learning rate of $1 e^{-5}$ and a batch size of four. The 3D segmentation models have a slower convergence such that the learning rate had to be increased to $1 e^{-4}$. Due to memory constraints, the batch size had to be set to one. The loss function used for training all networks was the Dice loss, based on the Dice coefficient described in the metrics section.

To evaluate the time necessary to execute the automatic segmentation pipeline in Fig. 1, full-resolution volumes were passed through the pipeline. The pipeline consists of reading the images from the file system, pre-processing, forward pass through the network, post-processing and writing the images to the file system. As the HR-pQCT scanner is not equipped with the hardware necessary for the inference of the network, the pipeline was executed on a dedicated workstation equipped with an Nvidia RTX2080 with 11 gigabytes of video memory. Since the forward pass through the network is non-iterative, the time to execute the pipeline does not depend on the difficulty of the case at hand. Both networks, the 2D U-Net and the 3D U-Net, were compared by passing 50 volumes sequentially through the respective pipelines and measuring mean and standard deviation (SD) of the execution time. The workstation for the network inference is separate from the HR-pQCT scanner. Therefore, the software necessary for the pipeline’s execution can be run constantly. This reduces the overhead of loading the software components for the inference of new patients. Since the 2D U-Net operates on single slices, to get the prediction of the second MC for the volume of a patient, the network iterates over all slices of the patient’s measurement. In contrast, the 3D U-Net is able to predict the second MC region with a single forward pass through the network.

#### Post-processing

Once the network is trained, the predictions of the second MC location for new patients had to be integrated into the clinical workflow. This is done by the post-processing pipeline described in the following.

First, the predictions of the left hands flipped in the pre-processing had to be flipped again along the y-axis in order to get a prediction consistent with the patient’s hand. In a few cases, the predictions for the second MC mask suffered from small disruptions inside the bone region. Since for the later vBMD measurements, a solid mask is required, the holes were removed using a morphological active contour step^35^. In the pre-processing, the input volume was reduced to the slices annotated by the expert. Since the prediction for the whole patient volume is required for the vBMD measurement, the dropped slices had to be recovered as a final step.

Using the HR-pQCT scanner software, a contour can be generated using the prediction volume (described in detail in the “Supplementary information” section). The resulting contour file can then be loaded into the scanner evaluation tool and checked by the technical assistants. In case the prediction is correct, the vBMD measurement tool can be executed.

#### Metrics

To assess the performance of the networks in the three phases, training, validation, and inference, certain metrics have to be defined to measure the quantitative outcome.

A very typical performance metric for segmentation networks is the Dice coefficient (DSC)^36–38^. The Dice coefficient compares the expert annotation $Y\in \{0;1\}^N$ with the prediction $X\in \{0;1\}^N$:

$$\begin{aligned} {\text {DSC}}(X, Y) = \frac{2 |X \cdot Y|}{|X| + |Y|}. \end{aligned}$$

With the corresponding Dice loss:

$$\begin{aligned} {\text {Dice}}_{\text {loss}}(X, Y) = 1 - {\text {DSC}}(X, Y). \end{aligned}$$

The pixel accuracy measures the percentage of correctly classified pixels:

$$\begin{aligned} {\text {Accuracy}}(X, Y) = \frac{\sum _{i=1}^{N} X(i) \cdot Y(i) + (1 - X(i)) \cdot (1 - Y(i))}{N}. \end{aligned}$$

The area under the receiver-operator-characteristic (AUROC) allows assessing the network’s capability to distinguish between two classes. To calculate the AUROC, the area under the ROC curve is measured. The ROC curve plots the true positive rate over the false positive rate for increasing threshold values for the prediction.

Finally, the intersection over union (IOU) is a widely used performance metric for segmentation models and is closely related to the DSC:

$$\begin{aligned} {\text {IOU}}(X, Y) = \frac{|X \cdot Y|}{|X| + |Y|}. \end{aligned}$$

#### Qualitative evaluation

To evaluate the predictions of the networks (see Fig. 4), an expert rating was performed. The expert annotator was presented with two contours placed on top of the HR-pQCT image. As the evaluation was performed at full resolution and for the whole patient volumes, the expert could scroll through all slices for each patient. Then, the expert was asked to classify the presented case into three classes. Either one of the two contours was chosen as the best one or none was chosen. In order to avoid a bias towards a specific contour color, for each new patient volume, the colors were randomly perturbed.

## Supplementary Information

Supplementary Information.

## Data Availability

In order to increase the repeatability of this work, we publish the code to train the network, run the inference for new images, and the integration into the clinical workflow. The code is available at https://github.com/lukasfolle/Automatic-vBMD-Measurements. Due to privacy regulations, neither the dataset nor the trained network parameters can be shared publicly.

## References

[1] McInnes IB, Schett G (2006). The pathogenesis of rheumatoid arthritis. N. Engl. J. Med..

[2] Ritchlin CT, Colbert RA, Gladman DD (2017). Psoriatic arthritis. N. Engl. J. Med..

[3] Schett, G. & Gravallese, E. Bone erosion in rheumatoid arthritis: Mechanisms, diagnosis and treatment. *Nat. Rev. Rheumatol.* **8, **656–664. 10.1038/nrrheum.2012.153 (2012). 10.1038/nrrheum.2012.153 PMC4096779 23007741

[4] Rau R, Lingg G, Wassenberg S, Schorn C, Scherer A (2005). Bildgebende verfahren in der rheumatologie: Konventionelle röntgendiagnostik bei der rheumatoiden arthritis. Zeitschrift fur Rheumatol..

[5] Werner D (2017). Early changes of the cortical micro-channel system in the bare area of the joints of patients with rheumatoid arthritis. Arthritis Rheumatol..

[6] Simon D (2017). Age- and sex-dependent changes of intra-articular cortical and trabecular bone structure and the effects of rheumatoid arthritis. J. Bone Miner. Res..

[7] Simon D (2018). A comparative analysis of articular bone in large cohort of patients with chronic inflammatory diseases of the joints, the gut and the skin. Bone.

[8] Peters M (2019). Prospective follow-up of cortical interruptions, bone density, and micro-structure detected on HRpQCT: A study in patients with rheumatoid arthritis and healthy subjects. Calcif. Tissue Int..

[9] Burghardt AJ (2010). A longitudinal HR-pQCT study of alendronate treatment in postmenopausal women with low bone density: Relations among density, cortical and trabecular microarchitecture, biomechanics, and bone turnover. J. Bone Miner. Res..

[10] Ostertag A (2014). Cortical measurements of the tibia from high resolution peripheral quantitative computed tomography images: A comparison with synchrotron radiation micro-computed tomography. Bone.

[11] Töpfer D, Finzel S, Museyko O, Schett G, Engelke K (2014). Segmentation and quantification of bone erosions in high-resolution peripheral quantitative computed tomography datasets of the metacarpophalangeal joints of patients with rheumatoid arthritis. Rheumatology (United Kingdom).

[12] Ching T (2018). Opportunities and obstacles for deep learning in biology and medicine. J. R. Soc. Interface..

[13] Klein A, Warszawski J, Hillengaß J, Maier-Hein KH (2019). Automatic bone segmentation in whole-body CT images. Int. J. Comput. Assist. Radiol. Surg..

[14] Benjamens S, Dhunnoo P, Meskó B (2020). The state of artificial intelligence-based FDA-approved medical devices and algorithms: an online database. NPJ Digit. Med..

[15] Bland JM, Altman DG (1986). Statistical methods for assessing agreement between two methods of clinical measurement. Lancet.

[16] Regensburger A (2015). A comparative analysis of magnetic resonance imaging and high-resolution peripheral quantitative computed tomography of the hand for the detection of erosion repair in rheumatoid arthritis. Rheumatology (United Kingdom).

[17] Burghardt AJ, Buie HR, Laib A, Majumdar S, Boyd SK (2010). Reproducibility of direct quantitative measures of cortical bone microarchitecture of the distal radius and tibia by HR-pQCT. Bone.

[18] Nishiyama, K. K. & Shane, E. Clinical imaging of bone microarchitecture with HR-pQCT. *Curr. Osteoporos. Rep.* **11**, 147–155. 10.1007/s11914-013-0142-7 (2013). 10.1007/s11914-013-0142-7 PMC4102136 23504496

[19] Whittier DE, Mudryk AN, Vandergaag ID, Burt LA, Boyd SK (2020). Optimizing HR-pQCT workflow: A comparison of bias and precision error for quantitative bone analysis. Osteoporos. Int..

[20] Valentinitsch A (2012). Automated threshold-independent cortex segmentation by 3D-texture analysis of HR-pQCT scans. Bone.

[21] Lauze FB, Hahn HK, Mori K (2019). Automatic detection and localization of bone erosion in hand HR-pQCT. Medical Imaging 2019: Computer-Aided Diagnosis.

[22] Figueiredo CP (2018). Methods for segmentation of rheumatoid arthritis bone erosions in high-resolution peripheral quantitative computed tomography (HR-pQCT). Semin. Arthritis Rheum..

[23] Włodarczyk J, Wojciechowski W, Czaplicka K, Urbanik A, Tabor Z (2015). Fast automated segmentation of wrist bones in magnetic resonance images. Comput. Biol. Med..

[24] Blavnsfeldt AG (2020). Effect of radiographic disease severity in high-resolution quantitative computed tomography assessment of metacarpophalangeal joint erosion and cysts. Int. J. Rheum. Dis..

[25] Erhan D (2010). Why does unsupervised pre-training help deep learning?. J. Mach. Learn. Res..

[26] Xie Y, Richmond D (2019). Pre-training on grayscale imagenet improves medical image classification. Lecture Notes Comput. Sci. (Including Subseries Lecture Notes Artif. Intell. Lecture Notes Bioinform.).

[27] Hesamian MH, Jia W, He X, Kennedy P (2019). Deep learning techniques for medical image segmentation: Achievements and challenges. J. Digit. Imaging.

[28] Sode M, Burghardt AJ, Pialat JB, Link TM, Majumdar S (2011). Quantitative characterization of subject motion in HR-pQCT images of the distal radius and tibia. Bone.

[29] Ronneberger, O., Fischer, P. & Brox, T. U-net: Convolutional networks for biomedical image segmentation. *Lecture Notes Comput. Sci. Including Subseries Lecture Notes Artif. Intell. Lecture Notes Bioinform.*10.1007/978-3-319-24574-4_28 (2015). arXiv:1505.04597.

[30] Yakubovskiy, P. Segmentation Models Pytorch. *GitHub Repos.* (2020).

[31] He, K., Zhang, X., Ren S. & Sun, J. Deep residual learning for image recognition. In: *Proceedings of the IEEE Computer Society Conference on Computer Vision and Pattern Recognition.* IEEE Computer Society 770–778. 10.1109/CVPR.2016.90 (2016).

[32] Russakovsky O (2015). ImageNet large scale visual recognition challenge. Int. J. Comput. Vis. (IJCV).

[33] Isensee, F., Kickingereder, P., Wick, W., Bendszus, M. & Maier-Hein, K. H. Brain tumor segmentation and radiomics survival prediction: Contribution to the BRATS 2017 challenge. In: *Lecture Notes in Computer Science (including subseries Lecture Notes in Artificial Intelligence and Lecture Notes in Bioinformatics)*. Springer Verlag. 287–297. 10.1007/978-3-319-75238-9_25 (2018).

[34] Maas AL, Hannun AY, Ng AY (2013). Rectifier Nonlinearities Improve Neural Network Acoustic Models.

[35] Marquez-Neila P, Baumela L, Alvarez L (2014). A morphological approach to curvature-based evolution of curves and surfaces. IEEE Trans. Pattern Anal. Mach. Intell..

[36] Milletari, F., Navab, N. & Ahmadi, S. A. V-Net: Fully convolutional neural networks for volumetric medical image segmentation. In: *Proceedings - 2016 4th International Conference on 3D Vision, 3DV**2016*. Institute of Electrical and Electronics Engineers Inc. 565–571. 10.1109/3DV.2016.79 (2016).

[37] Vesal, S., Ravikumar, N., Ellman, S. & Maier, A. Comparative analysis of unsupervised algorithms for breast MRI lesion segmentation. In: *Informatik aktuell*. Springer Berlin Heidelberg. 257–262. 10.1007/978-3-662-56537-7_68 (2018).

[38] Drozdzal, M., Vorontsov, E., Chartrand, G., Kadoury, S. & Pal, C. The importance of skip connections in biomedical image segmentation. in *Lecture Notes in Computer Science (including subseries Lecture Notes in Artificial Intelligence and Lecture Notes in Bioinformatics)*, Springer Verlag 2016. 179–187. 10.1007/978-3-319-46976-8_19 (2016).

