# Supplementary material for: Deep learning methods allow fully automated segmentation of metacarpal bones to quantify volumetric bone mineral density
Source: Sci Rep. 2021 May 6;11:9697. doi: 10.1038/s41598-021-89111-9 (PMC8102473; doi:10.1038/s41598-021-89111-9)
Supplement: Supplementary file 1 — Supplementary Information. [file 41598_2021_89111_MOESM1_ESM.pdf]

Deep Learning Methods Allow Fully Automated Segmentation of Metacarpal Bones to Quantify Volumetric Bone Mineral Density

Lukas Folle[1,*], Timo Meinderink[2,3], David Simon[2,3], Anna-Maria Liphardt[2,3], Gerhard Krönke[2,3], Georg Schett[2,3], Arnd Kleyer[2,3,+], and Andreas Maier[1]

[1]Pattern Recognition Lab - Computer Science, Friedrich-Alexander-Universität Erlangen-Nürnberg (FAU), Germany
[2]Department of Internal Medicine 3 - Rheumatology and Immunology, FAU Erlangen-Nürnberg and Universitätsklinikum Erlangen, Germany
[3]Deutsches Zentrum für Immuntherapie, FAU Erlangen-Nürnberg and Universitätsklinikum Erlangen, Germany
[+]arnd.kleyer@uk-erlangen.de [*]lukas.folle@fau.de

Supplementary information

vBMD results for the trabecular and cortical region

For completeness, the division of the average bone mineral density into the cortical area and the trabecular area is reported in the following.

The trabecular area using the manual pipeline had an average area of 96.57 mm^2 and 93.31 mm^2 for the automatic pipeline, and for the cortical area using the manual pipeline 14.76 mm^2 and 13.04 mm^2 for the automatic pipeline. For the cortical bone mineral density (Dcomp) the results of the manual pipeline were between 423.2 mg HA/cm^3 and 760.2 mg HA/cm^3 with a mean of 633.88 mg HA/cm^3 (SD 67.10) and for the automatic pipeline between 365.2 mg HA/cm^3 and 745.4 mg HA/cm^3 with a mean of 602.95 mg HA/cm^3 (SD 77.71). Finally, for the trabecular bone mineral density (Dtrab) the manual pipeline results were in the range from 112.1 mg HA/cm^3 to 239.8 mg HA/cm^3 with a mean of 183.50 mg HA/cm^3 (SD 28.92), and for the automatic pipeline in the range from 105.4 mg HA/cm^3 and 237.0 mg HA/cm^3 with a mean of 178.90 mg HA/cm^3 (SD 29.65).

Significant Pearson correlation for Dcomp with 0.997 ($p < 0.001$), and for the Dtrab with 0.999 ($p < 0.001$) was reached. Additionally, significant Spearman's-rank for Dcomp with 0.989 ($p < 0.001$), and for Dtrab with 0.996 ($p < 0.001$) was achieved. These correlations are demonstrated in the Bland-Altman plots in Figure 1. A skew of the differences for the Dcomp measurement in Figure 1 is noteworthy. As the cortical bone volume of the second MC attributes only approximately 12% to the total bone volume, the correct detection of this area is substantially harder than the trabecular bone volume, and thus for patients with increasing bone volume and bone mass, this error is reduced.

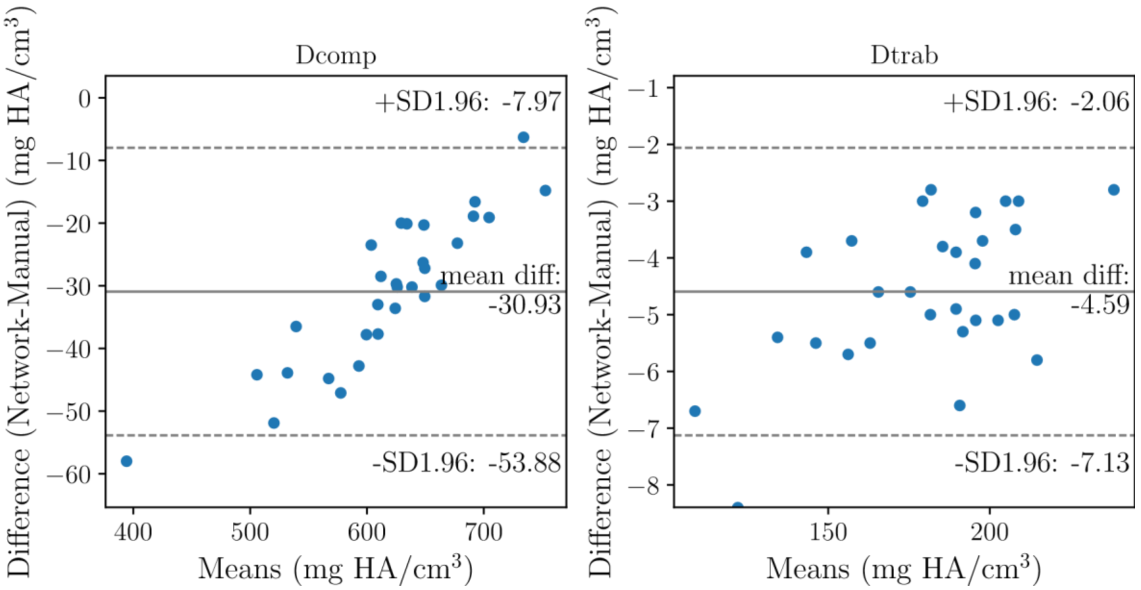

Figure 1. Bland-Altman plots of the agreement between manual and automatic pipeline for the cortical bone density (Dcomp), and the trabecular bone density (Dtrab).

File type conversion

To convert the contour file generated by the expert annotator, the following commands have to be executed in the image processing language using the workstation of the HR-pQCT manufacturer. First, the contour file has to be converted to a binary image file: /gobj_to_aim gobj_filename temp 0. Then, the header information of the binary image file has to be saved separately: /write_part temp file_output header binary. Finally, the binary image file is saved to the file system: /write temp aim_filename bin true.

All the previously generated files have to be transferred to the workstation running the neural networks. Once the networks predicted the location of the second MC, the resulting binary image file has to be converted to a contour file to enable the usage in the clinical workflow. First, the prediction is read from the file system: /read pred_aim_filename temp. Then, a type conversion is necessary for the last step: /convert_to_type temp temp char. Finally, the contour file can be generated using the converted image file: /togobj_from_aim temp gobj_filename.

Now the generated contour file can be viewed using the manufacturer's software and the vBMD measurement can be initiated.
